# Experimental Investigation of Nanolayered Silver and MXene Coatings for Enhanced Energy Efficiency in Compact Heat Exchangers

**DOI:** 10.1021/acsomega.6c05128

**Published:** 2026-07-29

**Authors:** Mehmet Mete Ozturk, Bahadır Doğan, Suat Pat

**Affiliations:** † Eskişehir Osmangazi University, Department of Aeronautical Engineering, Eskişehir TR-26480, Türkiye; ‡ Eskişehir Osmangazi University, Department of Mechanical Engineering, Eskişehir TR-26480, Türkiye; § Eskişehir Osmangazi University, Department of Physics, Eskişehir TR-26480, Türkiye

## Abstract

Energy efficiency remains a critical priority for industrial systems, striving to ensure operational sustainability and reduce environmental impacts. Because thermal management is central to industrial efficiency, optimizing compact heat exchangers through low-cost, minimal-impact surface improvements is a primary strategy for achieving substantial energy savings without altering existing manufacturing infrastructure. To address this, this study experimentally investigated nanolayered silver and MXene (V_2_AlC) coatings to improve the energy efficiency of compact heat exchangers. Nanomaterial coatings were applied to the aluminum surfaces of two distinct heat exchanger designs using thermionic vacuum arc technology. Experimental data revealed that depositing an initial silver layer enhanced the thermal performance by 13% for both heat exchangers. The addition of a second silver layer yielded a cumulative performance increase of 22% for HEX1 and 28% for HEX2. While the third silver layer introduced increased thermal resistance, depositing a fourth layer composed of MXene successfully induced a recovery in thermal performance, restoring high thermal conductivity, and maintaining superior system efficiency. The structural and morphological evaluations, alongside the thermal measurements, demonstrate that the strategic layer-by-layer application of these nanomaterials significantly improves the heat transfer rates. This enhancement leads to an overall reduction in energy consumption, ranging from 18% to 28%. Ultimately, these novel nanolayered architectures represent a highly effective intervention for optimizing thermal management, offering a practical, cost-effective solution for advancing green energy and low-consumption cooling devices.

## Graphical abstract

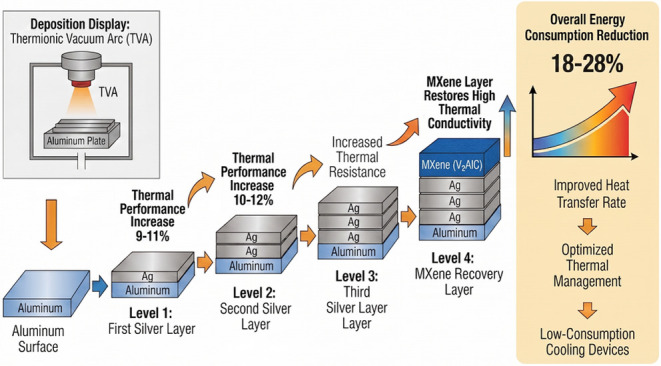

## 1 Introduction

The implementation of high-performance design innovations is often constrained by the inherent rigidity of existing industrial manufacturing infrastructures, while energy efficiency is a primary objective for conventional systems.^1^ A new design can achieve the best performance, but industrial manufacturing lines cannot be changed easily, which limits the implementation of such designs in practice. However, enhancing devices for minimal effect and low-cost improvement processes are needed for energy efficiency and energy-saving methods, such as upgrading insulation, using energy-efficient lighting, and implementing smart technology to monitor and reduce energy consumption.^2,3^ Furthermore, the long-term adoption of any innovative thermal technology necessitates a rigorous evaluation of its energy, economic, and environmental (EEE) impacts. As demonstrated in recent life cycle assessments of solar thermal applications, balancing these EEE considerations is essential to justify new sustainable implementations.^4^ Often viewed as the most economical form of renewable energy, energy efficiency and energy-saving measures provide critical utility for industrial applications such as mining, HVAC systems, data centers, and power electronics.^5,6^ New energy facilities require significantly more investment, whereas energy efficiency and energy savings represent the most cost-effective investments in energy. A marginal 1% enhancement in global energy efficiency translates into substantial economic dividends with the potential to reduce global expenditure by approximately $2 trillion USD annually. Furthermore, the capital efficacy of such initiatives is noteworthy, yielding an estimated return on investment of $3 to $5 for every dollar allocated. The realization of these economic and energy-saving dividends relies heavily on the technical optimization of thermal management systems, where heat exchangers play a pivotal role in maintaining the operational efficiency. In this context, this paper proposes a novel energy-saving method by using silver and MXene nanomaterial coatings to heat exchanger (HEX) surfaces that serve as a high-performance intervention to achieve these efficiency objectives.^7,8^

HEXs are fundamental thermal management components engineered to facilitate the transfer of thermal energy between two or more fluids via a solid interface, thereby preventing cross contamination. These devices are ubiquitous across various engineering sectors including air conditioning systems, automotive cooling, and microelectronics. A primary criterion for the structural classification of heat exchangers is their area density, defined as the ratio of the heat transfer surface area to the total. Systems exceeding a threshold of 700 m^2^/m^3^ are categorized as compact heat exchangers. This high area-to-volume ratio allows for significant thermal dissipation within constrained spatial envelopes, making them ideal for high-performance applications.^9,10^ In air-cooled applications, specifically those involving the thermal regulation of electronic components, data centers, transformers, air conditioners, refrigerators, automobiles, and electric vehicle engines, up to date, enhanced performance has been achieved, often optimized through the integration of extended surfaces (fins) on the air side. These geometries are designed to disrupt boundary layer development and increase turbulence, thereby enhancing the convective heat transfer coefficient. Beyond geometric optimization, the thermal conductivity of the heat exchanger material serves as a critical determinant of the overall thermal effectiveness. The synergy between high-conductivity materials (such as aluminum or copper alloys) and advanced fin geometries dictates the total thermal resistance of the system. The paper shows a new approach to reach an enhanced cooling mechanism and introduces a novel material to obtain the highest rate of energy efficiency. According to the literature, various materials such as phase change materials,^11^ magnetocaloric materials,^12^ boron nitride and aluminum nitride,^13^ and teflon-based coatings^14^ can be used to increase the heat transfer coefficient, heat dissipation coefficient, and overall thermal management of the system. Indeed, recent literature highlights that advanced selective coatings and material innovations offer substantial performance advantages, such as increasing thermal collection efficiency by up to 15% and serving as a critical factor in achieving commercial viability for next-generation thermal systems.^15^ The impact of copper coating on heat pipe performance is investigated across a wide range of coating thicknesses.^16^ Heating loads to determine its overall effect on thermal performance and electroless plating of Ni, Co, and Ag on plate heat exchangers is reported to evaluate how thermal and hydraulic properties are affected, and the findings demonstrate efficiency changes by comparing plated surfaces with plain samples.^17^

To elevate the thermal management capabilities of fluidic systems, nanoparticles and two-dimensional materials like MXenes are frequently dispersed as additives in base fluids. Recent investigations by Kumar and Shaik demonstrated that MXene-based nanofluids exhibit exceptional convective heat transfer performance, identifying them as highly innovative coolants.^18^ The intrinsic properties of MXenes, most notably their superior thermal conductivity, make them highly attractive for advanced thermal regulation.^19,20^ While the bulk of existing literature focuses on MXenes as liquid suspensions or generalized coating agents, the direct application of these materials as specialized surface coatings, particularly on complex geometries such as flat-tube configurations, remains underexplored due to the complexities of current manufacturing and deposition technologies. Consequently, direct MXene coating architectures represent a critical, high-performance frontier in optimizing heat transfer systems. Coating processes are simple and basic methods compared to the MXene or nanomaterial-added liquid cooling system. The use of oil as a cooling liquid leads to varying outcomes, resulting in numerous engineering problems. These coatings provide properties such as low friction and serve as dielectric media, among others. Nanoparticles and MXene powders can destroy the device’s performance in mixing approaches. But coating is just used over the outer surface, and the inner structure is still invariant. In this approach, coating is the useful choice for the enhanced cooling effect without changing the system’s working performance.^21–23^ MXene materials represent a significant innovation in science and technology, offering intriguing potential across various applications. While widely utilized in battery innovation, MXenes also present a compelling option for cooling mechanisms, though the specific configurations required to optimize performance remain to be clearly defined. These materials follow the general formula M_*n*+1_X*_n_* T_*x*_, where M is an earth transition metal, X is carbon or nitrogen, and T represents surface functional groups. MXenes are synthesized from Ti_2_AlC MAX phases using various techniques and can be deposited onto surfaces via physical vapor deposition (PVD) technology. In the field of thermal management, cooling studies continue to yield significant insights; however, a definitive material or process for extreme heat dissipation remains elusive. While MXene has emerged as a prominent novel candidate, silver-based solutions consistently demonstrate superior performance. Specifically, silver coatings have been extensively documented in thermal research,^24–27^ as silver-integrated materials substantially enhance heat dissipation in electronic applications. Furthermore, while carbon-based structures show promise, the synergy found in silver–carbon composites (utilizing graphene or graphite) currently delivers the most effective cooling results.

According to the literature, heat transfer was calculated to be five times more than that of conventional silver-coating materials. In Table 1, thermal conductivity of some materials are listed. Diamond and carbon compounds such as graphene have the largest thermal conductivity. One other important parameter is a heat dissipation coefficient, which is related to the heat passing through the medium from a hot surface. Investigating the literature, the heat dissipation coefficient of the carbon and nanocoated surface can increase to 3–10 times compared to the uncoated conventional material.^28,29^ Copper and aluminum dissipation coefficients are very close to each other, which conveys heat to liquid or gas media. As we know, surface properties play a critical role for the end surface temperature of the samples.^30^

**Table 1:** Thermal Conductivity of Some Materials

material	thermal conductivity (W/mK)
silver	424
copper	400
aluminum	237
diamond	900–2370
graphene	2000–5000
MXene	580–800

In this research, silver and MXene coatings forming the MAX phase were realized by a physical vapor deposition (PVD) technology, whose name is thermionic vacuum arc (TVA) thin-film coating device. TVA is an anodic plasma generator. PVD is known as MXene coating technology from the MAX phase. Two different HEXs were coated by PVD with layer-by-layer silver and MXene layers, and thermal performances were investigated. To present the thermal performances, all obtained and measured thermal results were given in the same plots. Coating layers were investigated with field emission scanning electron microscopy (FESEM) with energy dispersive spectroscopy (EDS) and atomic force microscopy (AFM) to show the nanostructures’ surface. Finally, it was found that the nanolayered coating increased the thermal performances of the HEX and decreased its energy consumption by about 18–28%. Concluded results will open a new research era about the thermal performances of the MXene and nanolayers. Probably, thermal investigations are the best practice for energy consumption, green energy, and low energy consumption devices.

## 2 Experimental Setup

The tested aluminum finned-tube heat exchangers are illustrated in Figure 1. Two distinct configurations, designated as HEX1 and HEX2, were evaluated; their detailed specifications are consolidated in Table 2. While both heat exchangers feature unique fin geometries and frontal areas, the primary differentiation between the two designs lies in the fin density and the air-side heat transfer area, dictated by the number of fin rows integrated between the serpentine flat tubes. HEX1 incorporates two fin rows between each serpentine tube, whereas HEX2 utilizes three. Consequently, this structural variation results in a different number of serpentine bends for each heat exchanger despite their identical frontal areas. A thermionic vacuum arc coating system was employed to coat the HEX with silver and MXene to investigate their effects on thermal performance. In addition to silver’s exceptionally high thermal conductivity, V2AlC MXene was incorporated into this study because its phases exhibit strong thermal anisotropy; characterized by high in-plane but restricted out-of-plane heat transfer, its direction-dependent thermal properties require thorough characterization for specific applications. Coating materials were bought by the commercial trade company. In the paper, HEXs were coated by TVA with silver layers and an MXene (V_2_AlC) layer. TVA is a high-purity coating and thin-film deposition system. The coating parameters are given in Table 3. The coating process was realized in high vacuum conditions of about 1 × 10^–5^ Torr. HEXs were well located in the vacuum vessel to coat with silver and MXene layers.

**Figure 1:**
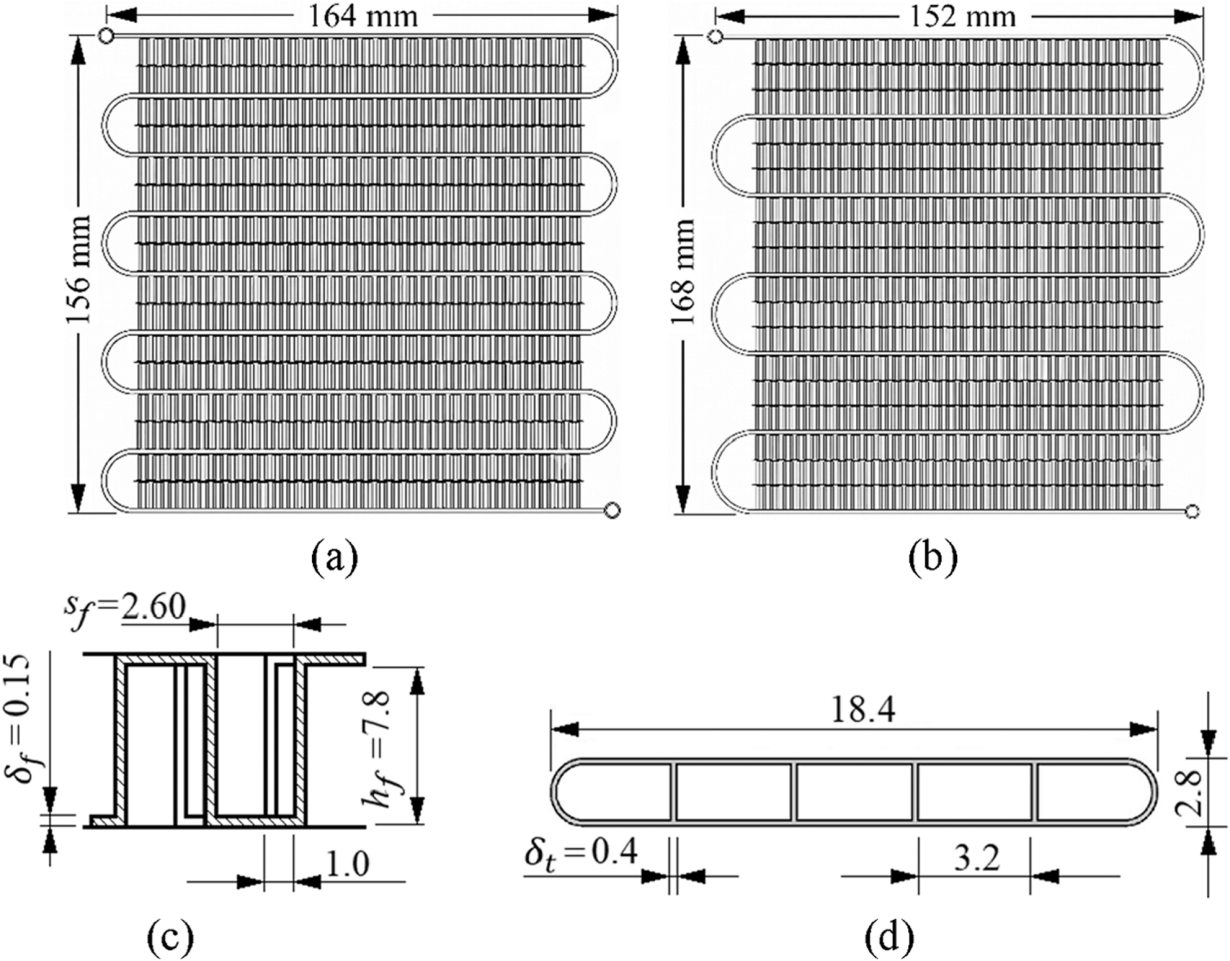
Considered HEXs. (a) HEX1, (b) HEX2, (c) details of fin, (d) details of flat tube.

**Table 2:** Specifications of the Condensers

parameter	symbol	unit	HEX1	HEX2
heat transfer area	*A*	m^2^	0.295	0.323
frontal area	*A*_fr_	m^2^	0.0256	0.0256
height × width	*H* × *W*	mm × mm	164 × 156	168 × 152
fin height	*h*_f_	mm	7.8	7.8
flow depth	*f*_d_	mm	17.15	17.15
flat-tube length	*l*_t_	m	1.55	1.25
fin spacing	*s*_f_	mm	2.6	2.6
flat-tube thickness	δ_t_	mm	0.40	0.40
fin thickness	δ_f_	mm	0.15	0.15
number of flat-tube passes	-	-	9	7
number of fin rows	*N*_f_	-	16	18
material	-	-	aluminum	aluminum

**Table 3:** Coating Parameters of Silver and MXene

deposition parameter	layer-1: silver	layer-2: silver	layer-3: silver	layer-4: MXene
vacuum pressure (torr)	1 × 10^–5^	1 × 10^–5^	1 × 10^–5^	1 × 10^–5^
filament current (A)	19	19	19	20
applied voltage (V)	600	600	600	800
voltage drops (V)	0	0	0	300
discharge current (mA)	500	500	500	300
duration (min)	2	2	2	2

In Figure 2, a schematic view of the TVA is illustrated. In the figure, the electron gun, materials, and electric connections are presented. An electron gun is made from a tungsten wire and a Wehnelt cylinder. The electron gun is to be connected as a cathode. The anode is an evaporation boat that contains the materials to be evaporated. In this study, silver slugs and MXene were placed into the evaporator boat separately. The electron gun was heated to 19 and 20 A for silver or MXene materials, respectively. Then, a high voltage was applied to the anode, which was about 600–800 V. After the materials melted, a plasma suddenly formed in the space between the electrodes. At this time, the bombardment current was suddenly increased to 500 mA. Deposition time was adjusted to 2 min for each deposition of silver or MXene. Operating under constant parameters for all experiments, a standardized layer-by-layer deposition method was utilized, with each 2 min coating cycle resulting in a consistent thickness increment of approximately 100 nm. The coating parameters are similar to those in the previous study.^28,31^

**Figure 2:**
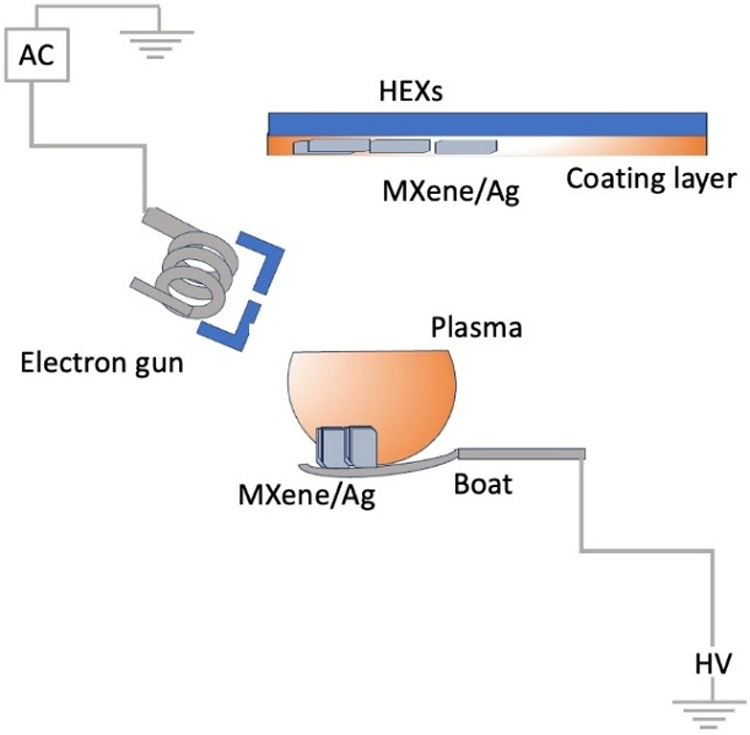
TVA coating system.

To determine the surface properties of the silver and MXene coating, field emission scanning electron microscopy (FESEM) and atomic force microscopy (AFM) were used. All coated layers were measured directly and FESEM images were obtained by Hitachi Regulus 8230 at room temperature. Energy dispersive spectroscopy is a chemical analysis technique and gives the ratio of coating layers. Ambios Q-Scope afm device was used for determining the surface properties, heights distribution of grains and average roughness of the coating layers. Scan rate was adjusted as 5 μm × 5 μm at 5 Hz. A silicon cantilever was used for scanning. AFM is a nondestructive surface analysis device.

The experimental setup utilizes a recirculating air tunnel and a closed-circuit water loop managed by a Programmable Logic Controller (PLC) as shown in Figure 3. The system regulates air velocities between 1.5–8.5 m/s and maintains temperatures within ± 0.5 °C using an R600a refrigeration cycle and a 6 kW heater. The test section, fabricated from plexiglass with a 190 mm × 190 mm cross-section, features flow straighteners to ensure uniform distribution around the centrally positioned heat exchangers. Instrumentation includes 20 T-type thermocouples for air temperature and an inlet flow meter. Water, maintained at a constant mass flow rate of 20 g/s (±3%), circulates through the heat exchanger via copper ports, with inlet and outlet temperatures monitored by direct-contact T-type thermocouples. To ensure a valid comparative analysis of coating effect, all experiments were conducted under standardized conditions: an air inlet temperature of 24 °C, a water inlet temperature of 43 °C, and water flow rate of 20 g/s.^32,33^ This standardized experimental protocol was strictly maintained for all evaluated heat exchangers, encompassing both the uncoated and coated configurations. Additionally, each experimental run was repeated three times to guarantee data reliability.

**Figure 3:**
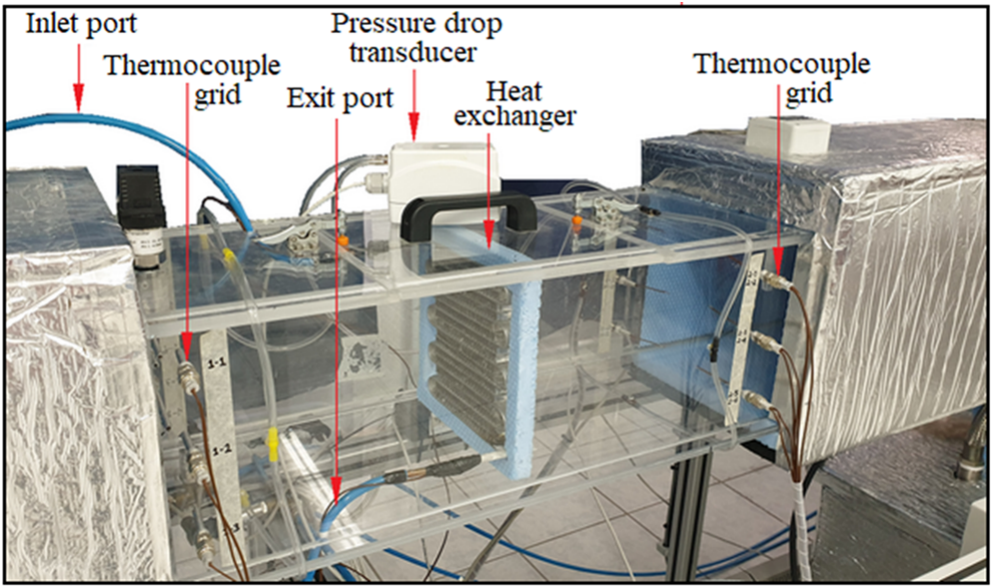
Thermal measurement setup.

The thermal capacities of the air and water flows are defined by eqs ([Disp-formula eq1]) and (2), while the system’s mean heat transfer rate is expressed in eq ([Disp-formula eq3]). To determine the overall heat transfer coefficient (*U*), the logarithmic mean temperature difference approach was employed according to eq ([Disp-formula eq4])

1 $${\mathrm{Q̇}}_{a}={ṁ}_{a}c_{p,a}\left(T_{a,o}-T_{a,i}\right)$$

2 $${\mathrm{Q̇}}_{w}={ṁ}_{w}c_{p,w}\left(T_{w,i}-T_{w,o}\right)$$

3 $$\mathrm{Q̇}=\left({\mathrm{Q̇}}_{a}+{\mathrm{Q̇}}_{w}\right)/2$$

4 $$U=\frac{\mathrm{Q̇}}{\mathrm{AF}\Delta T_{\mathrm{LM}}}$$

In this expression, *A* represents the total thermal exchange surface area, *F* denotes the crossflow correction factor, and Δ*T*_LM_ signifies the logarithmic temperature difference as calculated in eq ([Disp-formula eq5]).

5 $$\Delta T_{\mathrm{LM}}=\frac{\left(T_{w,o}-T_{a,i}\right)-\left(T_{w,i}-T_{a,o}\right)}{\ln\left[\left(T_{w,o}-T_{a,i}\right)/\left(T_{w,i}-T_{a,o}\right)\right]}$$

The thermal performance ratio, which evaluates the enhancement of the coated heat exchangers relative to the uncoated baseline, is defined by eq ([Disp-formula eq6]).

6 $$TPR=\frac{{\mathrm{Q̇}}_{\mathrm{coated}}}{{\mathrm{Q̇}}_{\mathrm{uncoated}}}$$

Uncertainties for the derived parameters were calculated using standard error propagation principles by considering eq ([Disp-formula eq7]). Table 4 details the specific uncertainties for all measured and calculated parameters.

7 $$U_{Y}=\sqrt{\sum_{i}{\left(\frac{\partial Y}{\partial X_{i}}\right)}^{2}U_{X_{i}}^{2}}$$

**Table 4:** Uncertainties for the Measured and Calculated Parameters

parameters	instrument	uncertainty
air temperature	T-type thermocouple	±0.4 °C
water temperature	T-type thermocouple	±0.4 °C
air velocity	velocity sensor—Siemens QVM62.1	±(0.2 m/s + 3% of FS)
water flow rate	volumetric flow meter—Saginomiya ELK-0501	±3.0% of FS
pressure drop of air	differential pressure sensor—Premasgard 1140	±3% of FS
heat transfer rate	calculated	2.19%
overall heat transfer coefficient	calculated	2.48%

## 3 Results and Discussion

Compared with bulk materials, the applied coating inherently possesses a vastly expanded surface area. As the heat dissipation coefficient is intrinsically linked to the surface area and structural topography, the coated surfaces achieve superior thermal dissipation. Therefore, a rigorous characterization of the sample surface properties is essential, making FESEM and AFM the most effective analytical devices for such investigations.^34,35^ In Figures 4 and 5, atomic force microscopy (AFM) images, field emission scanning electron microscopy (FESEM) images, and height distribution graphs of the coated layers are given.

**Figure 4:**
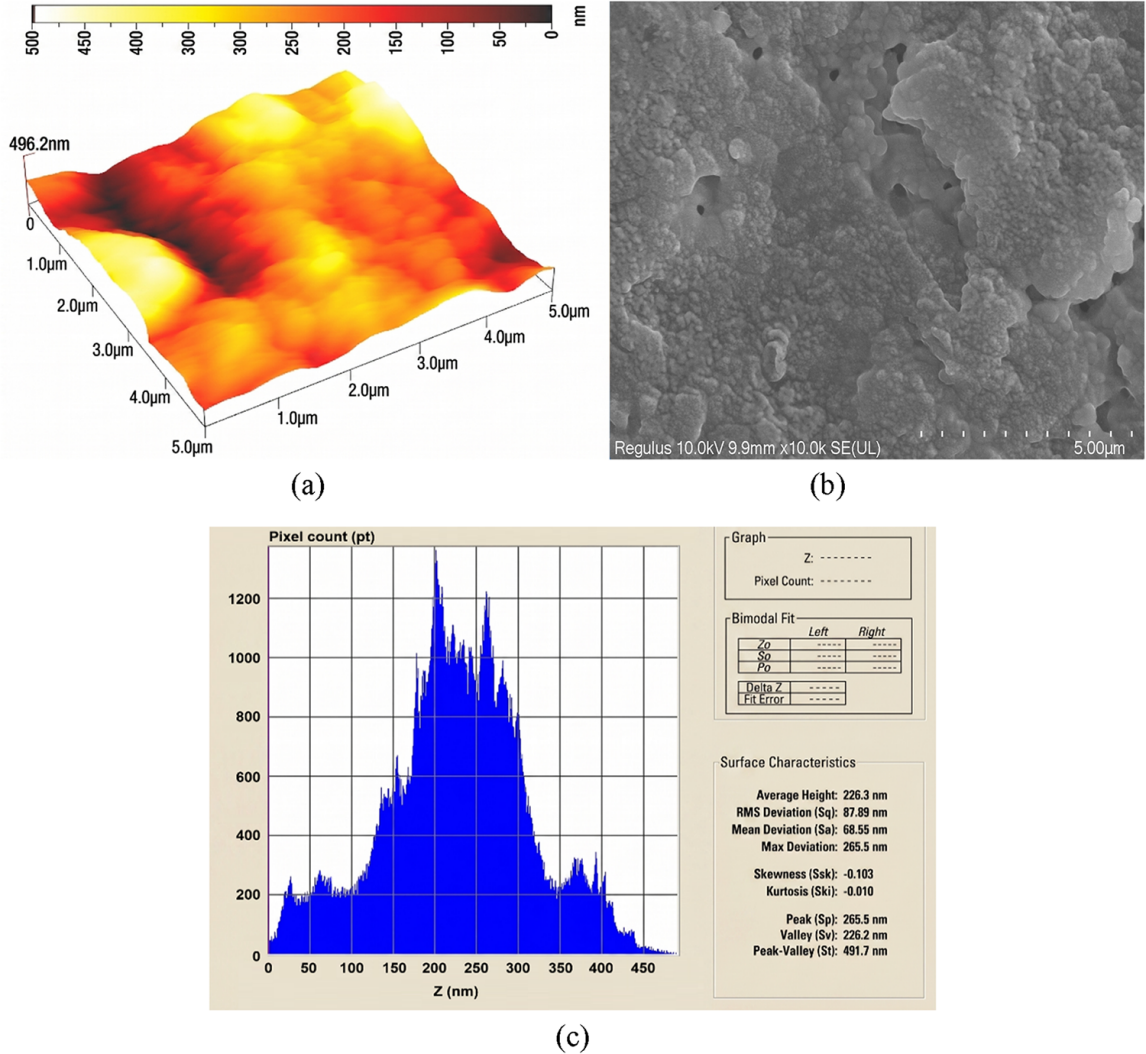
(a) AFM images, (b) FESEM images, and (c) height distribution graph of the silver coated (3 layers) on an aluminum sheet.

**Figure 5:**
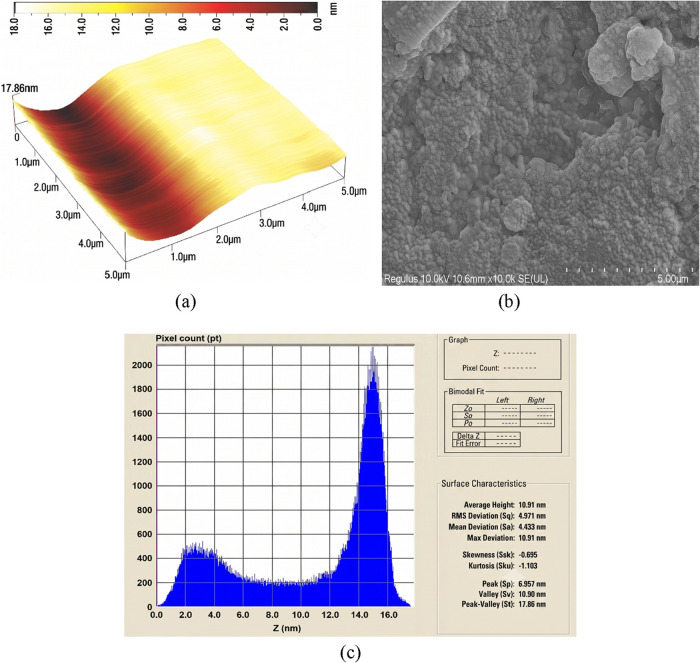
(a) AFM images, (b) FESEM images, and (c) height distribution graph of the MXene/silver (3 layers) coated on an aluminum sheet.

All images were obtained at room temperature, and no conductive layer was coated on the surface to enhance the images of the coated layers. The coating structures are silver-coated (3 layers) on aluminum sheets and MXene/silver-coated (3 layers) on aluminum sheets. According to the AFM images given in Figures 4a and 5a, the images of the coated surfaces in Figure 4a are seen more clearly, and grains or agglomerations can be detected. In Figure 5a, the top layer is the MXene layer, and the coating was started from the MAX phases of V_2_AlC powder. The maximum height of the surface is 496.2 nm. The AFM images were given in a 5 μm × 5 μm scale. The FESEM images are illustrated in Figures 4b and 5b for silver-coated (3 layers) on an aluminum sheet and MXene/silver (3 layers) coated on an aluminum sheet, respectively. The image scale was adjusted to 5 μm, and the FESEM scale and AFM scale are very close to each other. The images obtained were similar. The bright points in Figures 4b and 5b relate to the metal atoms. Surface structures are seen clearly for each image. The mean height of the grains/agglomerations was measured as about 200–250 nm for the silver coating on the aluminum sheet and 200 and 1400 nm for the MXene/silver (3 layers) coated on the aluminum sheet. According to the results, the silver coating has a maximum height of 200 nm and about 1400 nm for the MXene coating as seen in Figure 5c. The roughnesses of the coating layers are 71.9 and 4.51 nm for silver coated (3 layers) on aluminum sheet and MXene/silver (3 layers) coated on aluminum sheet, respectively. Skewness values were measured as −0.103 and −0.695 for the coatings. The surface images and parameters are in good harmony with the literature (Table 5).^28,36–41^

**Table 5:** EDX Results of the Coatings for HEX1 and HEX2

element	Wt% for HEXs	Wt% for HEXs
coating	silver (3 layers)	silver (3 layers)/MXene
C	-	47.22
Al	55.78	24.13
V	-	0.16
Ag	44.22	28.49
Total:	100.00	100.00

Since the goal of the heat exchanger is to achieve the targeted energy transfer via the tube extensions (fins) as the secondary fluid flows through the tubes, the performance of these components is measured by their heat-transfer rates under the thermal simulation conditions conducted in this study. The first pair of diagrams demonstrates the heat transfer rates of HEX1 and HEX2 under varying air velocities. The second pair pertains to the overall heat transfer coefficient, which represents the combined conductive and convective capabilities of the components and summarizes this relationship from a thermal resistance perspective.

As shown in Figure 6, the heat transfer rate increases as the frontal air velocity rises. This is directly correlated with the increase in the heat transfer coefficient caused by the higher airflow over the fins. In the diagrams, the lowest heat transfer rate corresponds to the uncoated HEX. When the HEX is coated with silver, the heat transfer capability gradually improves for both designs across all coating stages. The first layer of silver coating results in a 13% increase in heat transfer performance for both HEX designs. The thermal performance improves further with the second layer of silver; although its individual contribution is limited to 9%, the total cumulative improvement reaches approximately 23% compared to the plain HEX surface. This improvement likely stems from the combined effect of the silver film layers continuously breaking the boundary layer and a reduction in thermal resistance on the fin surfaces. The nanoscale roughness provided by the silver deposition disrupts the boundary layer, while the nanoscale thickness of the two silver layers helps reduce overall thermal resistance. However, with the third layer of silver, this increasing trend reverses. Although surface roughness might still increase, the added thermal resistance of the thicker film becomes the dominant factor, leading to a decline in heat transfer performance. To prevent further performance loss, MXene was deposited as a fourth layer. This MXene layer restores the heat transfer rate to the performance range of the second silver layer, falling slightly behind the second Ag layer in HEX1 and competing closely in HEX2.

**Figure 6:**
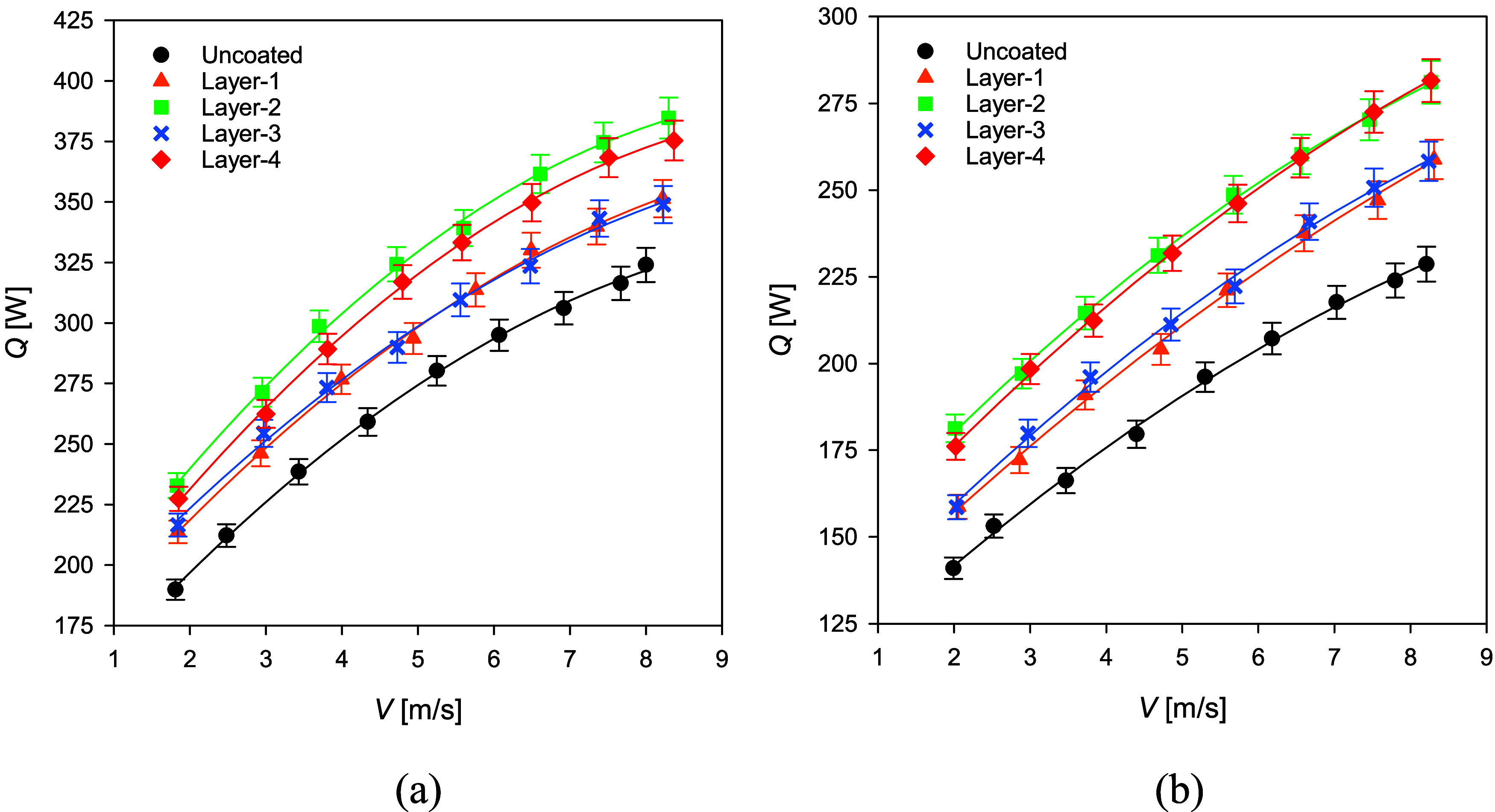
Impact of coating on the heat transfer rate vs frontal air velocity for (a) HEX1 and (b) HEX2.

The impact of thermal resistance on the HEX is represented by the overall heat transfer coefficient, which is detailed in Figure 7. Figure 7 presents the variation in the overall heat transfer coefficient (*U*) across different air velocities. As velocity increases, *U* also rises; this is because *U* is a function of the convective heat transfer coefficient (*h*), which is strongly dependent on air velocity. As described in previous sections, the overall heat transfer coefficient represents the total thermal resistance of the fin and tube structures and is defined by the relationship between the heat transfer rate and the temperature difference. Higher thermal resistance leads to a lower *U* and, consequently, a lower heat transfer rate (*Q*); conversely, a lower thermal resistance results in higher *U* and *Q*. When the aluminum fins and tubes are coated with silver (Ag), the thermal resistance decreases as the temperature differential drops. In both HEX designs (HEX1 and HEX2), the lowest *U* values are observed under uncoated conditions. As silver is deposited, the thermal resistance begins to fall, causing *U* to increase after the first and second layers. However, similar to previous observations, the *U* value declines with the deposition of the third Ag layer. This may be attributed to an increase in thermal resistance, as made evident by the rising temperature differentials. Following the deposition of the fourth layer (MXene), a recovery in the *U* values is observed in both designs (HEX1 and HEX2). Nevertheless, the performance trend remains similar and does not surpass those of the second Ag layer. It should also be noted that the curves for the different coating layers nearly overlap across most air velocities with only subtle differences visible, particularly between the third and fourth deposition layers.

**Figure 7:**
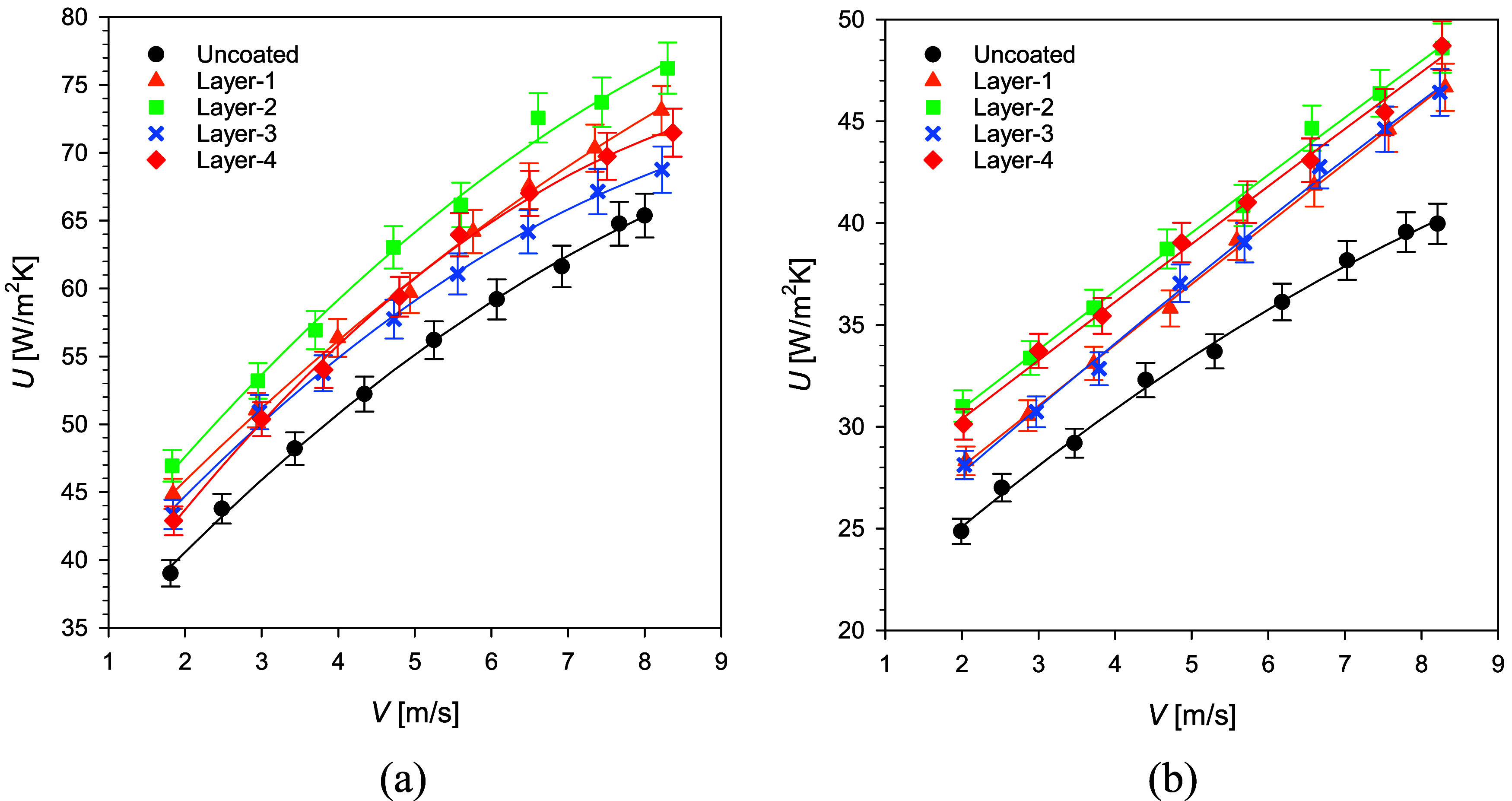
Impact of coating on the overall heat transfer coefficient vs frontal air velocity for (a) HEX1 and (b) HEX2.

The impact of coatings on the air-side pressure drop of the heat exchangers considered is shown on Figure 8. As the air velocity increases, the resulting increase in the air flow rate leads to higher flow resistance, which consequently causes a continuous rise in the pressure drop. As depicted in Figure 8a,b for HEX1 and HEX2, the pressure drop at the lowest considered air velocity is approximately 5 Pa, whereas this value reaches around 110–115 Pa at the highest air velocity. Furthermore, when examining the specific effects of the layer-by-layer coating process, the deviations observed for each subsequent layer, from the uncoated baseline through Layer-4, remain strictly within the 3% uncertainty limit of the differential pressure sensor. The primary reason for this outcome is that the cumulative thickening of approximately 400 nm on the fin surfaces, resulting from the application of four coating layers, is negligibly small compared to the fin pitch of 2.6 mm. Therefore, this microscopic geometric change does not introduce any substantial additional resistance to the overall airflow.

**Figure 8:**
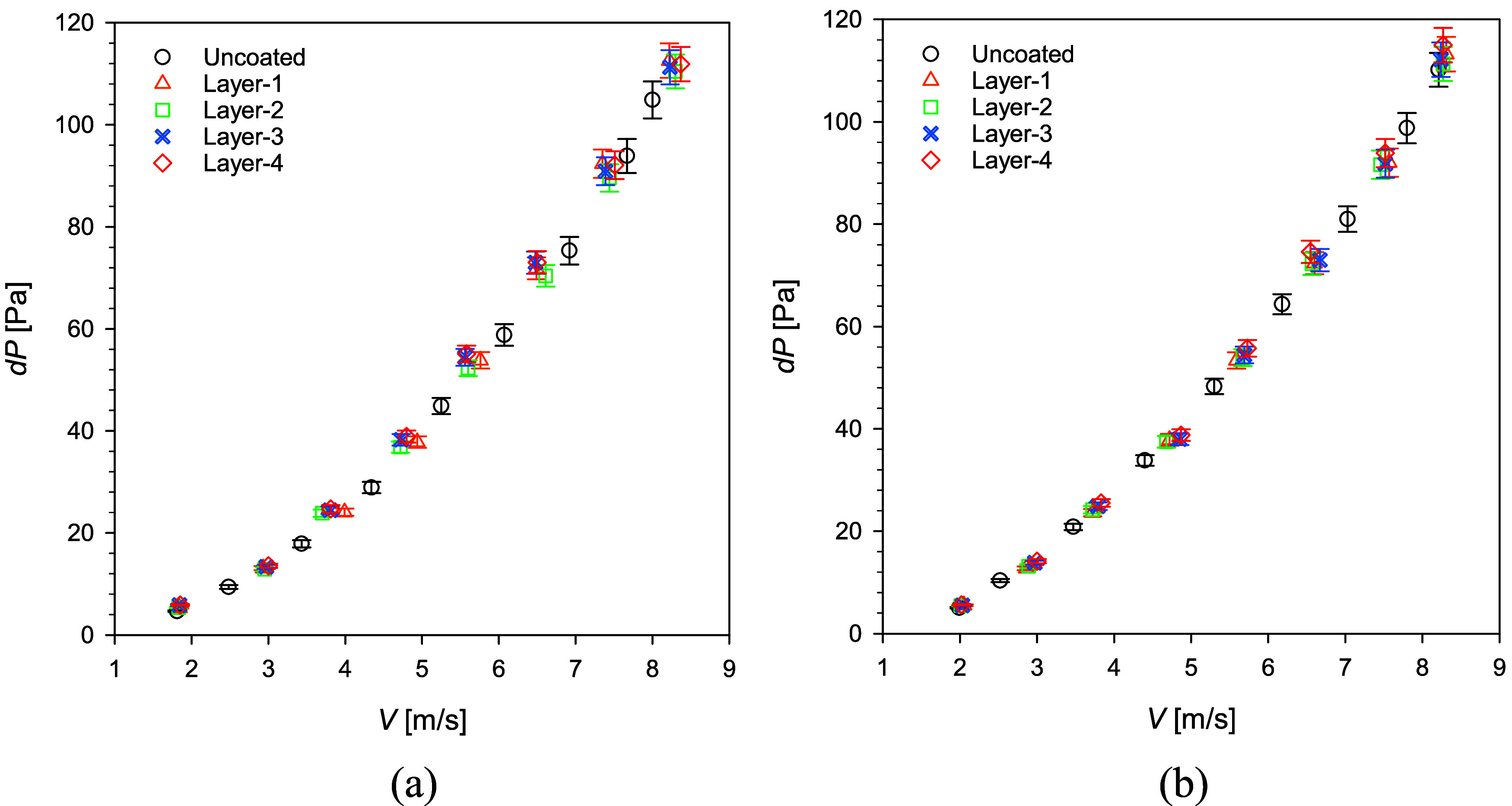
Impact of coating on the air side pressure drop vs frontal air velocity for (a) HEX1 and (b) HEX2.

While the contribution of the film layers to the heat transfer capability of the plain HEX designs was introduced in previous sections, the impacts of coating layers is more clearly observed through the deviation bars and curves in Figures 9 and 10. Figure 9 summarizes the deviation in contribution from one layer to the next with respect to varying frontal air velocity, while Figure 10 presents the overall cumulative impact. Figure 9 illustrates the deviation either increment or decrement of each layer relative to the previous one using a bar graph; orange represents the change from the plain HEX to the first layer, green compares the second layer to the first layer, blue shows the change from the second to the third layer, red demonstrates the change from the third to the fourth layer. In alignment with the findings in Figure 9, every layer except the third has a positive impact on the heat transfer capacity. While the first, second, and fourth layers all improve performance, the highest contribution is observed after the deposition of the second Ag layer. This trend is consistent across both HEX designs; excluding the third layer, heat transfer capacity typically increases by approximately 5–10% with each subsequent deposition. Literature indicates that while thermal conductivity may initially withstand thickness increments, it decreases rapidly beyond a critical point.^42^ This behavior underscores a delicate thermodynamic balance: the heat transfer enhancement driven by nanoscale surface roughness disrupting the boundary layer is eventually overcome by the simultaneously growing thermal resistance of the thicker film.

**Figure 9:**
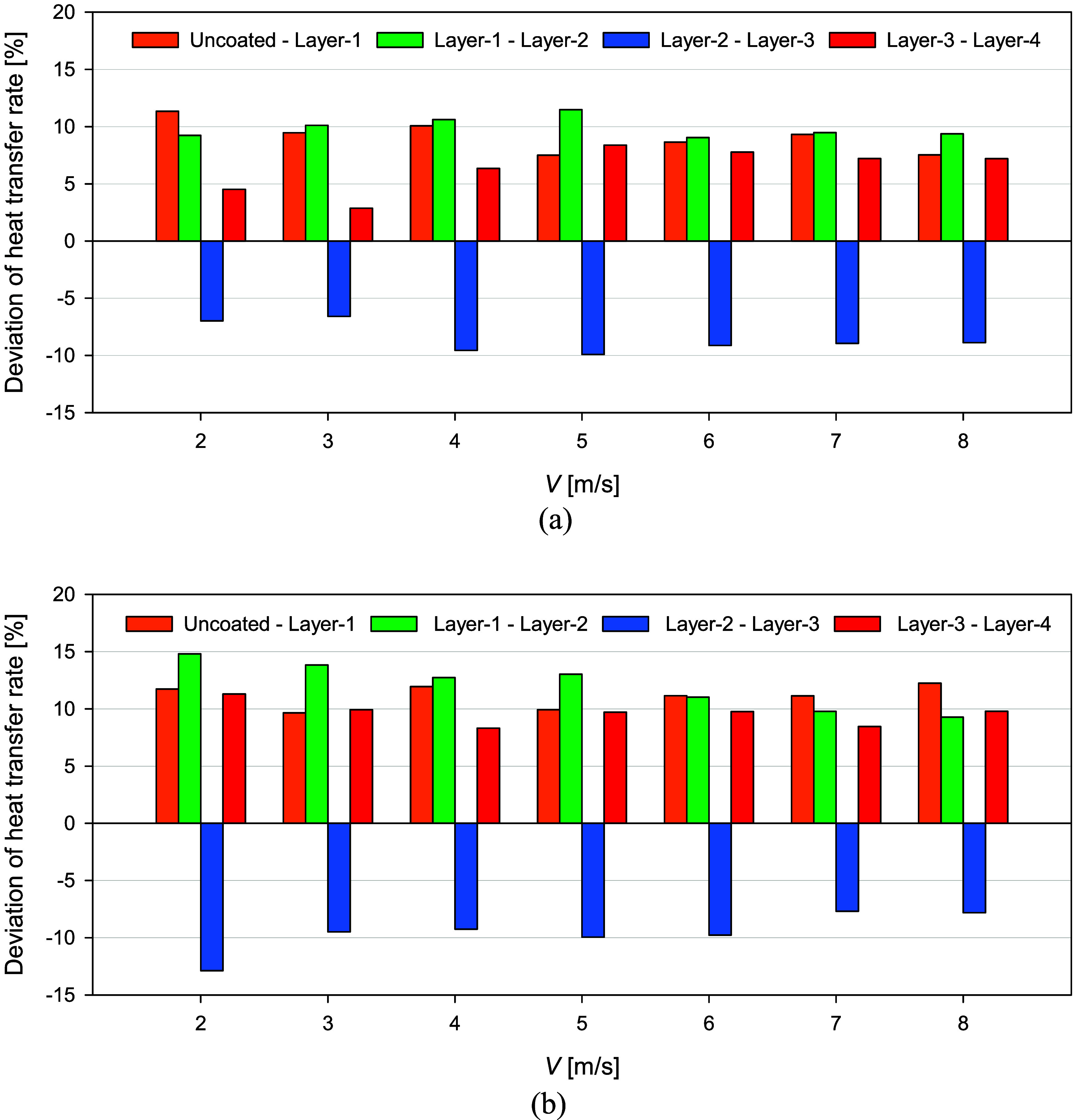
Impact of coating layer-by-layer on the heat transfer rate for HEX1 and HEX2.

**Figure 10:**
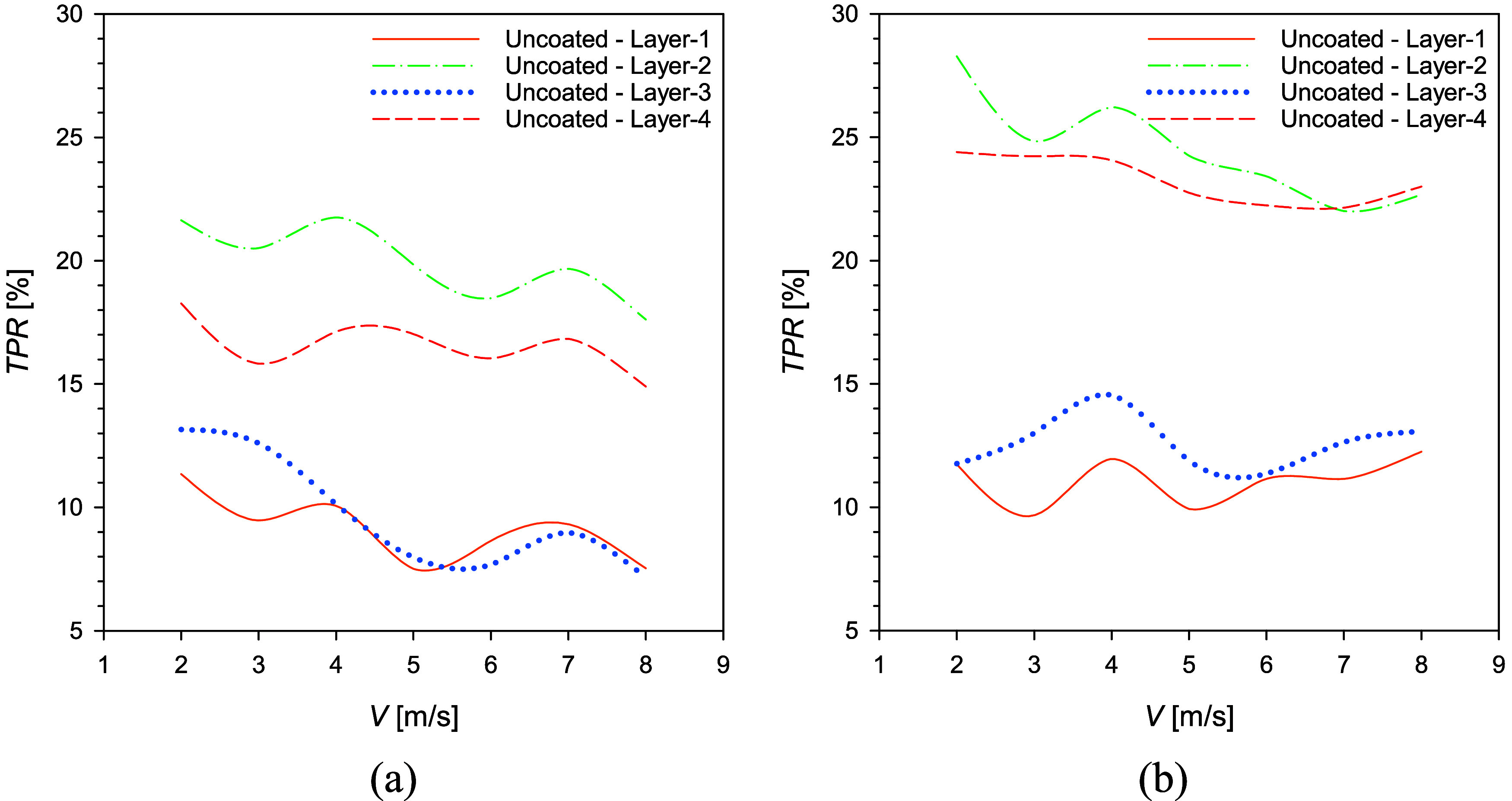
Thermal performance ratio (TPR) for (a) HEX1 and (b) HEX2.

As shown in Figure 10, the highest cumulative increment represented the thermal performance ratio (TPR) by occurring at low frontal velocities and begins to decrease as velocity increases across all layers. After the deposition of the first Ag layer, the heat transfer increment for HEX1 reaches 12% at low velocities and decreases slightly to 8% at the highest air velocity. For HEX2, the increment reaches a similar range but remains nearly constant regardless of changes in air velocity. When the second Ag layer is deposited, the heat-transfer capability of both HEX designs increases significantly, reaching 22% and 28%. These values then fall back to the 20% and 25% range, respectively, as air velocity increases. However, after the deposition of the third Ag layer, the heat-transfer ability of both HEXs drops below the performance of the second layer. Their contribution becomes almost equal to that of the first layer, which is attributed to the increased thermal resistance occurring across the HEX body. To mitigate this undesired effect and enhance the heat transfer capacity, MXene was deposited as the fourth layer. This resulted in a recovery of thermal performance. Compared to the third layer, this transition yielded a significant average increment of 6.3% for HEX1 and 9.5% for HEX2, although the improvement is not as high as that of the second layer, there is a significant increment compared to that of the third layer. In HEX2, the performance even approached that of the second layer at high air velocities.

## 4 Conclusions

This communication demonstrated the potential of nanolayered silver and MXene coatings, which are deposited via Thermionic Vacuum Arc (TVA) technology, to enhance the thermal performance of compact heat exchangers. After the application of a layer-by-layer deposition process, the research established a clear correlation between surface modification and energy performance improvement. As regards the outcomes:

- The deposition of the first two silver layers provided a substantial boost in the thermal performance, reaching a cumulative increase of 22% for HEX1 and 28% for HEX2.
- The thermal resistance bottleneck caused by the third silver layer was effectively offset by the fourth MXene layer. This final deposition induced a recovery effect, restoring high thermal conductivity and ensuring optimal system efficiency.
- The integration of these nanomaterials resulted in a significant reduction in energy consumption, ranging from 18% to 28% across the tested configurations.
- Morphological analysis via FESEM, EDS, and AFM confirmed that the PVD-based TVA process produced high-quality nanostructured surfaces capable of sustaining thermal improvements.

Ultimately, these findings confirm that the application of nanolayered surface modifications represents a promising and cost-effective approach to enhancing the industrial energy efficiency. The optimization of heat-transfer rates via silver and MXene coatings offers promising pathways for the development of next-generation thermal systems.

## Nomenclature

A: heat transfer area, m^2^
*A*_*fr*_: frontal area, m^2^
*c*_*p*,*a*_: specific heat of air, J/(kgK)
*c*_*p*,*w*_: specific heat of water, J/(kgK)
F: correction factor for crossflow heat exchangers
*f*_*d*_: flow depth, mm
H: height of the heat exchanger, mm
*h*_*f*_: fin height, mm
*l*_*t*_: tube length, m
*ṁ*_*a*_: mass flow rate of air, kg/s
*ṁ*_*w*_: mass flow rate of water, kg/s
*N*_*f*_: number of tube rows between tubes
Q̇: average heat transfer rate, W
*Q̇*_*a*_: air side heat transfer rate, W
*Q̇*_*w*_: water side heat transfer rate, W
*s*_*f*_: fin spacing, mm
*T*_*a*,*i*_: inlet temperature of air, K
*T*_*a*,*o*_: outlet temperature of air, K
*T*_*w*,*i*_: inlet temperature of water, K
*T*_*w*,*o*_: outlet temperature of water, K
U: overall heat transfer coefficient, W/(m^2^K)
W: width of the heat exchanger, mm

## Greek letters

δ_*f*_: fin thickness, mm
δ_*t*_: flat-tube thickness, mm

## Abbreviations

HEX: heat exchangers
TPR: thermal performance ratio
TVA: thermionic vacuum arc
